# Bioimaging structural signatures of the oomycete pathogen *Sclerospora graminicola* in pearl millet using different microscopic techniques

**DOI:** 10.1038/s41598-019-51477-2

**Published:** 2019-10-23

**Authors:** Hunthrike Shekar Shetty, Sharada Mysore Suryanarayan, Sudisha Jogaiah, Aditya Rao Shimoga Janakirama, Michael Hansen, Hans Jørgen Lyngs Jørgensen, Lam-Son Phan Tran

**Affiliations:** 1 Department of Biotechnology, University of Mysore, Manasagangothri, Mysore 570 006 India; 2 Department of Botany, University of Mysore, Manasagangothri, Mysore 570 006 India; 3 Laboratory of Plant Healthcare and Diagnostics, PG Department of Biotechnology and Microbiology, Karnatak University, Dharvad, Karnataka India; 4 Department of Plant and Environmental Sciences and Copenhagen Plant Science Centre, University of Copenhagen, Thorvaldsensvej 40, DK-1871 Frederiksberg C, Copenhagen Denmark; 5 Institute of Research and Development, Duy Tan University, 03 QuangTrung, Da Nang, Vietnam; 6 Stress Adaptation Research Unit, RIKEN Center for Sustainable Resource Science, 1-7-22 Suehiro-cho, Tsurmi-ku, Yokohama 230-0045 Japan

**Keywords:** Cell wall, Fluorescence imaging

## Abstract

In this case study, the mycelium growth of *Sclerospora graminicola* in the infected tissues of pearl millet and the process of sporulation and liberation of sporangia and zoospores were observed using four different microscopic techniques. The cotton blue-stained samples observed under light microscope revealed the formation of zoospores with germ tubes, appressoria and initiation of haustorium into the host cells, while the environmental scanning electron microscopy showed the rapid emergence of sporangiophores with dispersed sporangia around the stomata. For fluorescence microscopy, the infected leaf samples were stained with Fluorescent Brightener 28 and Calcofluor White, which react with β-glucans present in the mycelial walls, sporangiophores and sporangia. Calcoflour White was found to be the most suitable for studying the structural morphology of the pathogen. Therefore, samples observed by confocal laser scanning microscopy (CLSM) were pre-treated with Calcofluor White, as well as with Syto-13 that can stain the cell nuclei. Among the four microscopic techniques, CLSM is ideal for observing live host-pathogen interaction and studying the developmental processes of the pathogen in the host tissues. The use of different microscopic bioimaging techniques to study pathogenesis will enhance our understanding of the morphological features and development of the infectious propagules in the host.

## Introduction

Many oomycete plant pathogens cause a considerable loss in food crops, including cereals, legumes, oil seeds, vegetables, and fruit crops^1^. The oomycetes are a diverse class of fungus-like eukaryotic microorganisms that share common morphological features with the true fungi^1^. Cellulose is a major cell wall component in oomycetes, whereas chitin is a major cell wall component of true fungi^2,3^. However, oomycetes often possess chitin synthases that are activated during the morphogenesis of young hyphae and mycelia^4^. The hyphal and mycelial structures of oomycetes are diploid during their vegetative growth stage, whereas most fungi are haploid (e.g., ascomycetes) or dikaryotic (e.g., basidiomycetes), even though exceptions exist^5^. The cells of the oomycetes can be morphologically distinguished from true fungi as their mitochondrial cristae are tubular, whereas the cells of true fungi are flattened^4^.

Pearl millet [*Pennisetum glaucum* (L.) R.Br.] is a vital cereal crop mainly cultivated in the arid and semi-arid regions of the world, as it is tolerant to drought and high temperatures^6^. Downy mildew disease, caused by *Sclerospora graminicola* (Sacc.) J. Schröt, is one of the major diseases of pearl millet, causing approximately 10–80% of the annual grain yield loss^7,8^. Thines *et al*.^9^ recognized 20 genera of downy mildews of which eight belong to cereal downy mildews. Among these eight, *S. graminicola* is an obligate biotrophic pathogen^9^. The primary inoculum of *S. graminicola* is oospores that are capable of surviving in diseased plant residues and soil^9^. Sporangia/zoospores are secondary sources of inoculum, originating from the infected leaves of host plants, and becoming air-borne which can spread to new susceptible hosts^9^.

**Figure 1:**
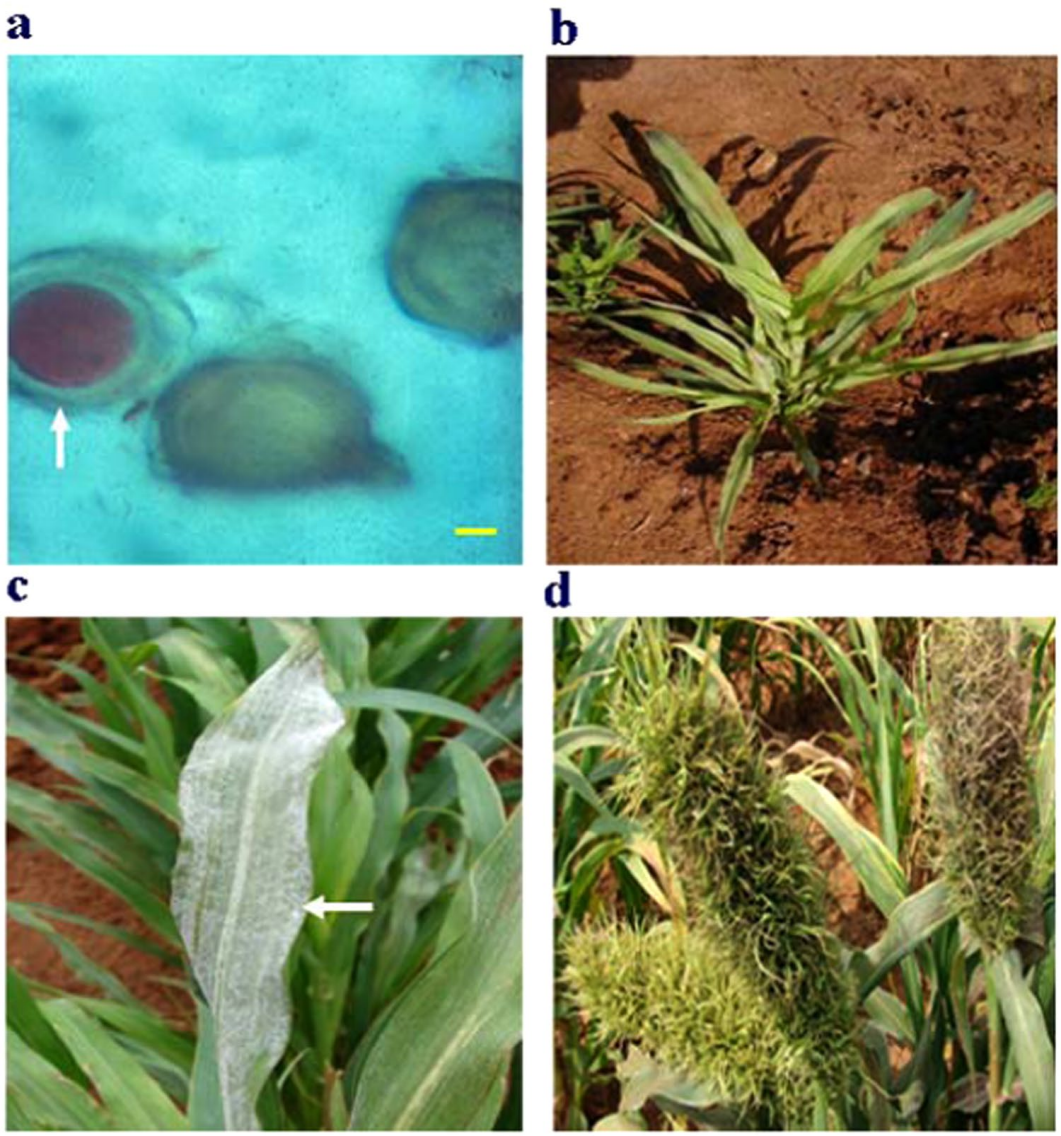
Sexual and asexual reproduction of *Sclerospora graminicola*. (**a**) 2,3,5-triphenyltetrazolium chloride-stained oospores of *S. graminicola*. Viable oospores were red-stained (white arrow), whereas non-viable spores were not stained as examined under a compound light microscope. Scale bar: 5 µm. (**b**) Typical downy mildew symptoms of pearl millet in the field at University of Mysore, India, showing asexual chlorotic stunted growth after 20 days of sowing of seeds coated with *S. graminicola* oospores. (**c**) Infected leaves with asexual sporulation of *S. graminicola* showed visible growth of the pathogen as indicated by the white-colored layer or white cottony growth (white arrow) on the lower surface of the leaves after 20 days of sowing. (**d**) Malformed panicles or green ear sexual sporulation of disease was formed after 56 days of sowing.

Fourteen grass species are recognized as hosts of *S. graminicola* strains with high host specificity observed among the pathogens^10,11^. The pathogens invade the hosts systemically, and infected leaves normally show yellowing or chlorosis along the veins of the leaves^12^ (Fig. 1). When the pathogen colonizes the inflorescence (panicle), the floral organs turn into leaf-like structures, and this abnormal development is termed phyllody^13^. Phyllody is the reason why the disease is referred to as ‘witch’s broom’, ‘crazy top’ or ‘green ear disease’^14^. These symptoms are common among the many host crop species, including pearl millet, infected by downy mildew pathogens belonging to the genera *Peronosclerospora*, *Sclerospora* and *Sclerophthora*^13,15^.

Bioimaging plays an important role in studies of fungal biology and plant pathology by allowing *per se* elucidation of cellular structures in living cells^16^. Recent advanced in fluorescence laser microscopic techniques, such as multiphoton microscopy and confocal microscopy, are new emerging areas for cellular exploration^17^. In addition, the applications of genetically encoded molecular markers, such as Green Fluorescent Protein (GFP)^18^, Fluorescence Resonance Energy Transfer (FRET) and *in situ* hybridization along with the various microscopic techniques have shown a great potential for investigating host-pathogen interactions^19^. Furthermore, Erukhimovitch *et al*.^20^ used Fourier Transform InfraRed (FT-IR) microscopy for early and rapid detection of *Colletotrichum coccodes*, the cause of black dot disease of potato (*Solanum tuberosum*). Bioimaging offers possibilities for gaining insights into host-pathogen interactions, which cannot be availed by using conventional techniques. Research in plant pathology is greatly influenced by newer advancements in techniques and imaging devices that can be used in combination with newer fluorescence techniques.

In the present investigation, mycelial structures and asexual reproduction of *S. graminicola* was examined for the first time in infected leaves using light, fluorescence, confocal, and environmental scanning electron microscopic techniques in combination with various stains. We aimed to test the suitability and simplicity of different microscopic techniques in identifying the developmental stages of the asexual structures of *S. graminicola* so that scientists can select the most appropriate approach(es) to obtain information on the host resistance and susceptibility mechanisms, such as papillae and callose formation, and molecular understanding of the host-pathogen interactions.

## Results

### *S. graminicola* oospore viability assay by light microscopy, and symptoms of *S. graminicola*-infected pearl millet

Prior to the seed coating, the viability of oospores used in the coating was tested by staining with 2,3,5-triphenyltetrazolium chloride (TTC) and examined under light microscope. Results showed that not all the oospores were viable. Specifically, when being observed under a compound light microscope oospores stained in red were viable, whereas non-stained oospores were not viable (Fig. 1a). Pearl millet seeds coated with the pathogen oospores were germinated 48 h after sowing and showed the typical asexual symptoms of downy mildew like leaf chlorosis and stunted growth at day 20 after sowing under field conditions (Fig. 1b). The white cottony growth symptoms occurred on the lower surface of infected leaves after 20 days of sowing (Fig. 1c). The disease symptoms continued until the panicle stage after 56 days of sowing, resulting in malformation of the floral structure into leafy structure that represents the sexual stage of pathogen (Fig. 1d).

### Light microscopy of pathogen structures in infected leaves

**Figure 2:**
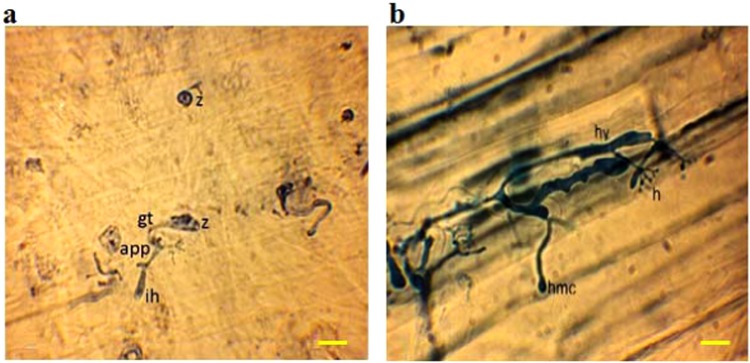
Light microscopy of initial asexual stages of *Sclerospora graminicola* infection on pearl millet leaves after inoculation of zoospores and staining with cotton blue. (**a**) Infection site on lower surface of pearl millet leaves observed under a compound light microscope showing germinated wall-less zoospores (z) with germ tube (gt), appressorium (app) and initiation of haustorium (ih) of *S. graminicola* after 3 h of inoculation. (**b**) *S. graminicola* surface mycelium from which haustorial mother cells (hmc), hyphae (hy) and haustoria (h) were developed inside the mesophyll cells, after the pathogen penetration into the epidermal cells of the host was initiated at 2th day, and continued to colonize the internal pearl millet tissues 4 days after being challenge-inoculated with the pathogen. Scale bar: 5 µm.

Infected leaves with asexual sporulation of *S. graminicola* exhibited intensive growth of the pathogen on the lower surface of the leaves. Such infected leaves subsequently showed the production of sporangiophore primordia, sporangiophores, sporangia and zoospores. Examination of infected lower leaf tissues from zoospores-infected seedlings stained with cotton blue under a compound light microscope revealed zoospore germination, as well as the initial stages of pathogen structures such as formation of germ tubes, appressoria and vesicles after 3 h of *S. graminicola* inoculation (Fig. 2a). After penetration, the induced hyphae were further developed into haustorial mother cells and other structures. The hyphae and haustoria penetrated the epidermal and mesophyll cells of the host within 2-days, and persistently colonized the pearl millet internal tissues following the challenge-inoculation with the pathogen (Fig. 2b).

### Fluorescence microscopy of live pathogen structures

**Figure 3:**
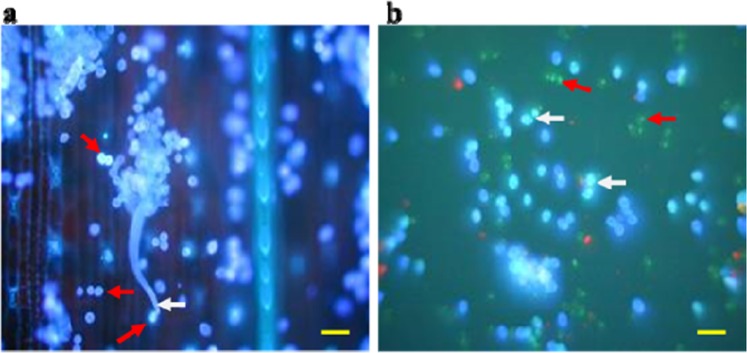
Fluorescence microscopy of asexual sporangiophores, sporangia and wall-less zoospores of *Sclerospora graminicola* after staining the infected pearl millet leaves with Calcoflour White at day 4 after inoculation. (**a**) A sporangiophore are seen emerging from a stoma (white arrow indicates basal cell of sporangiophore), and sporangia are scattered over the lower leaf surface (red arrow). (**b**) Sporangia (white arrow) and wall-less zoospores (red arrows). Scale bar: 5 µm.

To observe the various features of *S. graminicola* developing after its penetration into the plant cells, the infected leaf samples were stained with Fluorescent Brightener 28 and Calcofluor White, which are known to react with β-glucans present in the mycelial walls, sporangiophores, and sporangia. Our results revealed that Calcoflour White was the most suitable for studying the structural morphology of the pathogen. As shown in the representative images of staining with Calcofluor White, this chemical enabled us to clearly distinguish the features that have cell wall like mycelia, sporangiophores emerging from a stoma, and sporangia scattering over the leaf surface (Fig. 3a) from the wall-less zoospores (Fig. 3b).

### Confocal laser scanning microscopy (CLSM) of live pathogen structures

**Figure 4:**
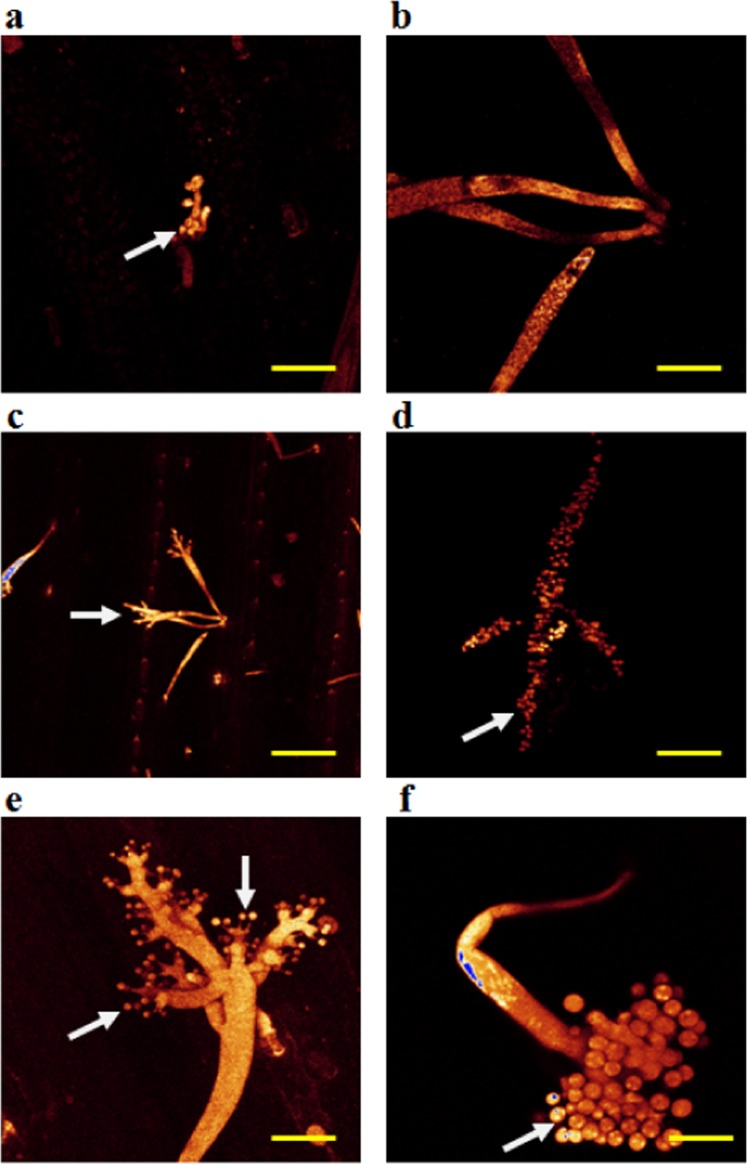
Confocal laser scanning microscopy showing asexual sporulation of *Sclerospora graminicola* after staining the infected pearl millet leaves with Syto-13 at day 4 after inoculation. (**a–c**) Sporangiophore primordia emerging through stomata on lower surface of an infected leaf. (**d**) Young sporangiophore showing sterigmata on which sporangia are developing. (**e**) Mature sporangiophore with fully developed sporangia. (**f**) Sporangiophore primordium migrating to the growing tips of the aseptate structure. Scale bars: 20 µm.

CLSM was used to study the asexual sporulation of *S. graminicola* in infected pearl millet leaves after staining with Syto-13 and Calcofluor White stains. Syto-13 only stained nucleic acids, making the multinucleate conditions of mycelia, sporangiophores and sporangia very clear (Fig. 4a–f). Sporangiophore primordia emerging through stomata in a Syto-13-stained infected leaf were observed in Fig. 4a–c, whereas a sporangiophore with sterigmata on which sporangia were developed could be noted in Fig. 4d, and a mature sporangiophore with fully grown sporangia could be seen in Fig. 4e. Additionally, Fig. 4f clearly showed a sporangiophore primordium with nuclei migrating to the growing tips of the aseptate structure after staining the infected pearl millet leaves with Syto-13.

**Figure 5:**
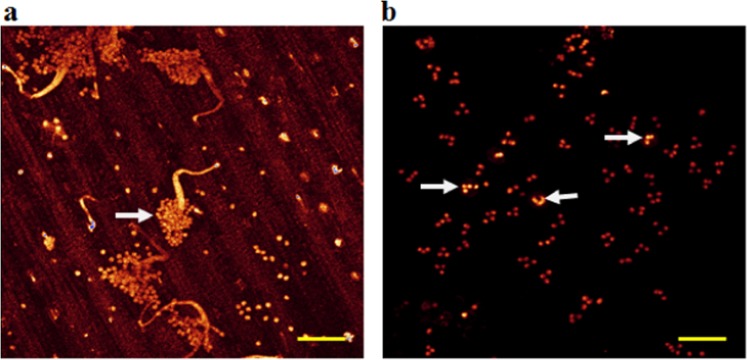
Confocal laser scanning microscopy showing asexual sporangiophores and sporangia after staining the infected pearl millet leaves with Syto-13 at day 4 after inoculation. (**a**) Sporangiophores and released sporangia of *Sclerospora graminicola* on the lower surface of an infected pearl millet leaf. (**b**) Sporangia releasing zoospores on the lower surface of the infected pearl millet leaf during sporulation. Fluorescent dots in groups of 3–5 are the nuclei in the sporangia stained with Syto-13. The stain is specific to nuclei. Scale bars: 20 µm.

Syto-13-staining of *S. graminicola*-infected pearl millet leaves indicated sporangiophores and sporangia released on the lower surface of infected leaves (Fig. 5a). Furthermore, sporangia, which released zoospores on the lower surface of the infected leaves during sporulation, were also stained and visualized with Syto-13. Each sporangium with 3–5 nuclei reacting with the stain Syto-13 showed liberated mononucleate zoospores (Fig. 5b). In addition, Syto-13 also induced staining in the cytoplasm of sporangia, but with less intensity, indicating that nucleic acids were present in the cytoplasm to some extent (Fig. 5b).

**Figure 6:**
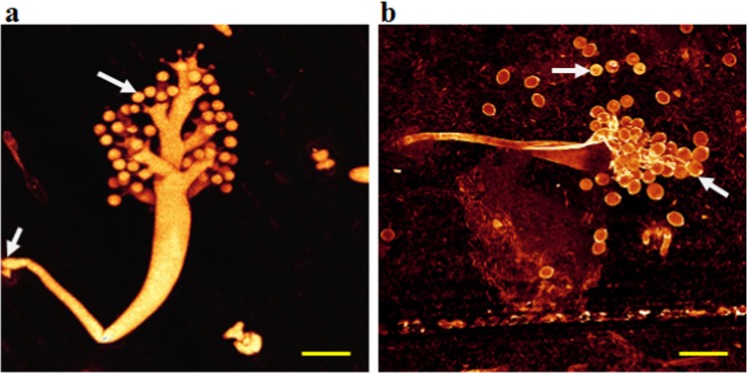
Confocal laser scanning microscopy of sporulation of *Sclerospora graminicola* showing asexual structures after staining the infected pearl millet leaves with Calcoflour White at day 4 after inoculation. (**a**) Sporangiophore on the lower leaf surface of an infected leaf. Arrow indicates basal cell of the sporangiophore. (**b**) Young sporangiophore on the lower leaf surfaceof an infected leaf. Arrow indicates the position of the operculum (opening) for release of zoospores in a sporangium. Scale bars: 20 µm.

Calcofluor White bound to *S. graminicola* cell wall, resulting in yellow/orange fluorescence, when it was examined by the CLSM. Figure 6a showed a CLSM image of a *S. graminicola* sporangiophore on an infected pearl millet leaf, with the basal cell of the sporangiophore. Furthermore, Fig. 6b presented sporangia on an infected pearl millet leaf, where the position of the operculum (opening) for zoospore release could be noted.

### Environmental scanning electron microscopy (ESEM) of live pathogen structures

**Figure 7:**
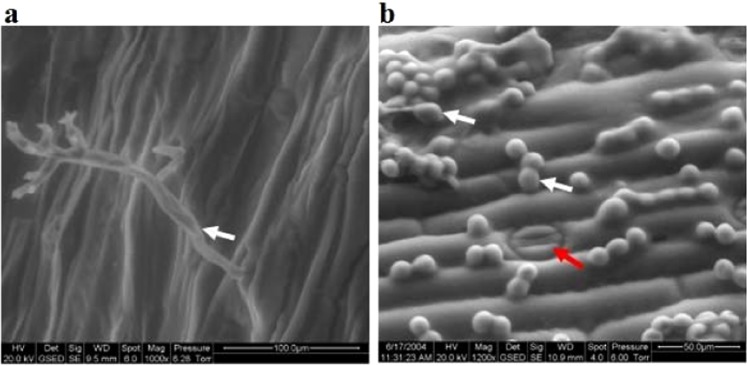
Environmental scanning electron micrographs of the lower surface of a *Sclerospora graminicola*-infected pearl millet leaf showing *S. graminicola* asexual structures at day 4 after inoculation. (**a**) Sporangiophore (white arrow) emerging through a stoma. Scale bar: 1000 µm. (**b**) Sporangia deposited on the leaf surface (white arrows). Red arrow indicates sporangia germinating with germ tube around a stoma. Scale bar: 500 µm.

The CLSM study was compared with another scanning technique, namely ESEM. In contrast to the conventional SEM, the ESEM allowed us to study fresh hydrated materials^21^. When infected pearl millet leaves were examined using ESEM, the leaf surface showed the emergence of sporangiophores (Fig. 7a), and subsequently the dispersed sporangia (Fig. 7b). ESEM was a quick and convenient method to examine the activity of *S. graminicola* structures on the infected leaf surface within 30 min without requiring any special staining or treatment prior to the microscopic observation^22^. A large number of sporangia deposited on the leaf surface were observed, and some were beginning to germinate (Fig. 7a,b). Many sporangia were deposited around the stomata, and the germ tubes of the sporangia often grew directly toward the stomata (Fig. 7b).

## Discussion

In the present investigation, the downy mildew pathogen *S. graminicola* of pearl millet was examined by using light microscopy, fluorescence laser microscopy, CLSM, and ESEM. For light microscopy, lower surface of the infected leaf tissues was processed, and then examined to study the early morphological features of the infection structures like wall-less zoospores, germ tubes, appressoria, and haustoria of *S. graminicola* after 3 h of inoculation (Fig. 2). This was time consuming, but relevant to study the details of interal pathogen structures. However, for external structures, further studies were carried out to observe live specimens without any tissue preparation (Figs 3–7).

The availability of various microscopic methods is critical to advance our understanding of the morphological features of different plant pathogens, and serves the basis for understanding of the interactions between hosts and pathogens at cellular level. Oomycete plant pathogens are unique in their morphological, biochemical, cytological and histological structures. Oomycetes are multinucleate with cell wall consisting of β-1,3- and β-1,6-glucans^21^. Studies of the intimate association between hosts and pathogens at cellular level and elucidation of the roles of different host and pathogen genotypes can shed light on understanding of the nature of disease resistance and advance our understanding of the host-pathogen interactions^23^. In the present report, the use of different types of microscopy to study pathogenesis and morphological features has enhanced our understanding of the morphological features of the infective propagules of the *S. graminicola* pathogen. Previously, Weston & Weber^24^ described morphological features of the pathogen *S. graminicola* in detail by drawing the sporangiophores and sporangia based on the data observed with a normal light microscope. In a separate research, the histology-based light microscopic analysis of pearl millet-*S. graminicola* interaction was investigated by Sharada *et al*.^25^. The authors demonstrated that the observation of developmental processes of asexual structural features of the pathogen by microscope allowed them to identify and predict the response of pearl millet seedlings to downy mildew disease, which was in good correlation with the results of disease incidence obtained under field conditions. Furthermore, the same authors also observed less haustoria and more papillae in the highly resistant pearl millet lines, while the reverse tendency for the highly susceptible lines^25^. In literature, there appear to be no references that provide a clear photomicrograph using either light or fluorescence microscopy or CLSM or ESEM. Therefore, this report provides the first photographic details of the *S. graminicola* pathogen in relation with the host pearl millet.

The use of fluorescence microscopy in studying plant-pathogen interactions complements the application of bright-field microscopy^17^. In the present study, it was observed that the fluorescence stains Fluorescent Brightener 28 and Calcoflour White reacted with β-glucans of the mycelial cell wall, sporangiophores and sporangia, while the wall-less zoospores did not (Fig. 3b). In our observation, Calcoflour White stain enabled us to elucidate the asexual structural development of the pathogen better than the Fluorescent Brightener 28 stain; and thus, photographs were taken (Fig. 3). Hansen *et al*.^26^ studied *Thanatephorus cucumeris* (syn. *Rhizoctonia solani*) hyphal structures and performed cytological studies on the oomycete *Pythium oligandrum*. In the present study of the *S. graminicola* oomycete, the nuclear cytology is clear, with a large number of nuclei in the hyphae and in the asexual reproductive structures sporangiophores and sporangia. Monheit *et al*.^27^ studied fungal cytology using Calcofluor White 28, and the results revealed that detection of the fungal pathogen by the combination of light and fluorescence laser microscopy was very effective compared with that of light microscopy alone. In addition, a KOH-Aniline Blue technique for fluorescence staining of fungi in association with plant tissues was developed by Hood & Shew^28^. However, even though the technique provided documentation of plant-fungal interactions, characteristics of host colonization, fungal reproduction and detection of inoculum, this procedure required extensive preparation for tissue clearing, which is time-consuming and may disrupt delicate pathogen structures.

The potential of microscopic techniques as investigation tools in plant-pathogen interactions has increased, and there is an increasing demand for using the fluorescent probes to study the structure of living cells. In addition, fluoresent labelling of specific components in cells can be generated without any manipulation of experiment. The applications of such techniques in studying the plant-pathogen interactions by locating the infection structures involved in the cellular alteration, defensive secretory processes and signaling pathways in susceptible and resistant host cells have been well documented^29,30^.

In general, it is not so easy to capture good images of cellular components labelled with fluorescent probes in isolated flat cells, including fungal hyphae. Furthermore, it is a challenging task to obtain high-resolution quality images of cellular components at various depths in plant cells, especially in those inside the plant organs. For instance, in the case of leaf samples, it is normally possible to visualize fluorescing structures in the epidermal cells present along the side of the leaf closest to the target objective^31–33^. However, it is very difficult to get clear images of structures associated with the mesophyll cells. Chloroplasts and other organelles often autofluoresce, and this will result in overlap with the emission spectra of the fluorochrome marker proteins^34^. The use of multiphoton lasers with increased penetration and small excitation volume may allow us to address this problem^35^. Computer-aided fluorescent microscopy, which uses higher light reflection, delivers better contrast and resolution of the host tissues and of the pathogen than conventional fluorescence microscopy^36^. In addition, computer processing can generate three-dimentional images of different structures of *S. graminicola* by assembling stacks of two-dimentional images^37^.

Recently, various techniques have been improved by Minker *et al*.^38^ for semi-automated confocal imaging of fungal pathogens on plants. Techniques and methods were optimized for sample fixation, optical clearing, species-specific fluorescence staining and image processing with reference to pathogenesis in maize tissues by the fungal pathogens *Exserohilum turcicum, Bipolaris maydis*, and *Cercospora zeae-maydis*^38,39^. CLSM is a new advancement in light microscopy, which mostly explores the latest laser, imaging and computational technologies that enable biologists to vizualize a distinctive form of cell and/or subcellular components^38^. The blur-free images of thick specimens can also be obtained by using computer tools and fluorescent light reflection at various depths. It is mainly advantageous for examining thick specimens or host-pathogen interactions where the pathogen is fixed deeply within the host tissue^36^. In the present study, CLSM provided optical sectioning of thick specimens at high resolution images of infected leaf tissues at various depths. Safeeulla^40^ used Feulgen stain to detect the nuclei in the mycelia, sporangia and zoospores of *S. graminicola* in pearl millet. However, the staining procedure is laborious and time consuming, whereas using Syto-13 is a very simple and convenient approach when compared with Feulgen staining. Overall, the CLSM combined with Syto-13 staining provides excellent opportunities for studying the live host-pathogen interaction with minimum disturbance of cellular developmental processes. Future studies involving immuno-cytolocalization of functional proteins and metabolites in pearl millet infected with *S. graminicola* may further enhance our understanding of the host-pathogen interaction during pathogenesis.

The emerging sporangiophores and the dispersed sporangia were observed by ESEM (Fig. 7). The quick and convenient procedure of ESEM, which could produce results within 30 min without any previous preparations, helped in examining the activity of *S. graminicola* infection structures in infected pearl millet leaves (Fig. 7). The capacity to view the fixed samples with minimum preparations has made this technique more advantageous and unique. Specimens can be observed directly, without drying and coatings. Pressure variations in drying units have been known to spoil and alter delicate biological materials^41,42^. Moreover, it reduces the preparation time, and its ability to scan directly the biological specimens enables soft specimens to be viewed with less mechanical damage^42^. Only a few studies have used ESEM to produce images of *S. graminicola*^43,44^. A clear image of the sporulating structures of pathogen on the infected leaves will give a better idea about its development and role in the epidemiology of the disease development under field conditions.

Taken together, the fluorescence microscopy allows us to capture high-resolution quality images of cellular components at various depths in plant cells, especially in those inside the plant organs^31,32,36^. The ESEM helps in understanding the sporangial deposition on the young leaf surface for forecasting the disease epidemiology and development^43^. In case of CLSM, the host cell response to the disease and the pathogen developmental processes at the early stage during the host-path interaction can be visualized^36,38^.

The advances in image processing technology and the assimilation of cellular and molecular approaches with advanced microscopy techniques have revolutionized the studies of cytological research. In this avenue, the studies by Hardham & Blackman^45^ on the position of plant receptors involved in *Phytophthora* spp. detection, and mechanisms of basal defence were demonstrated. Drake *et al*.^46^ extended the application of state-of-the-art methodologies, such as Synchrotron Radiation X-ray Tomographic Microscopy (SRXTM), Secondary Ion Mass Spectrometry (SIMS) and Time-of-Flight (ToF) SIMS, to enable comprehensive newer way to characterize fungi.

With the advances in bioimaging and the use of genetically encoded marker molecules like GFP, much attention will be given in future towards studies of living cells and materials with reduced requirements of fixed materials. Bioimaging offers better possibilities for studying host-pathogen interactions, which cannot be availed by using conventional techniques. In future, plant pathology will be profoundly influenced by such innovative bioimaging techniques, as well as the availability of genetic approaches and tools that can be used in an integrative manner with newer fluorescence techniques.

## Methods

### Host and pathogen

The downy mildew susceptible pearl millet cultivar 7042S was obtained from International Crop Research Institute for Semi Arid Tropics, Hyderabad, India. The oospores of *S. graminicola* were collected from the downy mildew infected plot at the University of Mysore, India.

### Oospore viability test

The presence of oospores in the seed samples was determined by the washing method, and the viability of the oospores was assessed by the 2,3,5-triphenyltetrazolium chloride test (TTC) as described by Shetty *et al*.^47^.

### Seed coating and generation of zoospores of *S. graminicola*

Seeds of the cultivar 7042S were sprinkled with sterile water, and the wetted seeds were coated manually with powdered oospores of *S. graminicola* for 30 min. The coated seeds were sown in the downy mildew infected plot at the University of Mysore. After eight days of sowing, germinated seedlings were observed for typical asexual disease symptoms in the form of chlorosis in the emerging leaves, stunted growth, white cottony growth on lower surface of leaves and necrosis on leaf tips. The symptomatic plants were regularly monitored up to the panicle stage (54–60-days of sowing) to evaluate the sexual stage of the pathogen. Such infected young leaves from 8-days old plants were harvested and kept for incubation in the evening and observed for sporulation during the early morning hours of the next day. Sporulation of the pathogen was also observed on the lower surface of infected leaves, including sporangiophore primordia as well as fully grown sporangiophores and sporangia. From such sporangia, 3–5 zoospores were liberated in water. The zoospore load was measured using a haemocytometer and adjusted to approximately 10^5^ zoospores mL^−1^, which was then used as an inoculum source.

### Plant treatment and tissue clearing for light microscopic studies

Twenty-five seeds of the cultivar 7042S were placed in six inch diameter Petri dishes lined with three layer of moistened blotting discs and incubated in growth chamber as explained above. The three-day old germinated seedlings were inoculated with *S. graminicola* by applying the asexual zoospore suspension (10^5^ zoospores mL^−1^) into the leaf whorls^48^. The inoculated seedlings were transered to 12 inch diameter pots containing the soil mixture ‘Weibulls Enhetsjord’ (K Jord, W. Weibull AB, Landskrona, Sweden) with all the required nutrients. Seedlings were raised in a growth chamber with alternative cycles of 12 h/12 h light and darkness. Light source was provided by fluorescent tubes (Osram L 36 W/11- 860 Lumilux plus Eco Daylight, Osram GmbH, Augsburg, Germany, 200 µE m^−2^ s^−1^). The temperature and humidity in the growth chamber were programmed to 25 °C/50–60% in day and 20 °C/80–90% during night, respectively. The infected seedlings were then harvested at different time intervals, namely 1, 3, 6, 12, 24, 48, and 96 h after inoculation, and fixed in a mixture of acetic acid and alcohol (1:3, v/v). The fixed seedlings were then soaked in 3% (w/v) NaOH for 30 min at 60 °C, and were then rinsed thoroughly in running distilled water for 30 min to remove the traces of NaOH. Subsequently, the treated seedlings were dried at room temperature and transferred to warm cotton blue [0.2% (w/v)] for 2 h to ensure uniform staining^25^.

### Light microscopy of live pathogen structures

Microscopic slides were prepared from live pathogen materials obtained from infected leaves harvested at different time intervals, namely 1, 3, 6, 12, 24, 48, and 96 h post-inoculation as previously described^40,48^. For light microscopy, the samples were stained using 0.2% cotton blue and were directly observed under a compound light microscope. The motile asexual sporangia releasing zoospores and penetration of pathogen propagules in the host tissues were directly observed using a Wild Leitz microscope (Wild MPS 46/52 Photoautomat, Switzerland).

### Stain preparation

The stain solutions used were:

- 0.03% (w/v) Fluorescent Brightener 28 (F6258, Sigma) in 0.2% phosphate buffer (w/v, pH 8.0)^49^.
- 3 µLmL^−1^Syto-13 (Molecular Probes)^26^.
- 0.1% (w/v) Calcoflour White (Polysciences) in distilled water containing 4,4’-bis-[4-anilino-bis-diethyl-amino-triazin-2-xylanimo-2,2′-stilbene disulfonic acid]^27^.

Fluorescent Brightener 28 was applied for 30 min, whereas Calcoflour White was applied for 1 min and Syto-13 for 15 min. For fluorescent microscopic observation, samples were prepared using Fluorescent Brightener 28 and Calcofluor White. For CLSM studies, samples were stained with Syto-13 and with Calcofluor White.

### Fluorescence microscopy of live pathogen structures

The infected leaves were harvested after 4 days of inoculation and were made to sporulate on Perspex plates as explained above and treated with each of the three stains for different durations. Microscopic slides were prepared with live asexual pathogen materials (sporangiophores, sporangia, and zoospores) obtained from infected leaves at 4th day post-inoculation. Sporangiophores, sporangia, and zoospores were observed using a fluorescence light microscope (Olympus BX60, Denmark). The filter used for Fluorescence Brightener 28 had excitation of 330–385 nm, dichroicmirror DM 400 nm and barrier filter >420 nm). Calcofluor White-stained samples was observed under UV light (excitation 365 nm and emission ∼440 nm, and DAPI (4′,6-diamidino-2-phenylindole) filter set was used).

### Confocal laser scanning microscopy

The interaction between plant and pathogen was monitored by CLSM using infected leaves of 4 days post-inoculation. The asexual structures, including sporangiophores, sporangia and zoospores of *S. graminicola*, were prepared and treated with two stains as described above, and were then observed by CLSM following the previously described method^26^. A confocal laser scanning microscope (TCS4d, Leica Laser Technik GmbH, Heidelberg, Germany) was used for detecting the fluorescence signals from stained pathogen structures. The instrument was assembled with an argon/krypton laser. The filter used for Syto-13 had excitation wavelength of 488 nm, beam splitter of double dichroic 488 and 568 nm and barrier filter of 515–545 nm.

### Environmental scanning electron microscopy

Infected leaves (4 days post-inoculation) showing asexual sporulation were directly observed using an environmental scanning electron microscope (FEI Quanta 200 ESEM, ThermoFisher Scientific, Waltham, Massachusetts, USA) without any prior staining or treatment. A Peltier stage maintained the temperature at 4 °C with a water vapour pressure of about 0.813 kPa (6.1 Torr) and the relative humidity approximately 100%. Secondary electrons released from the specimen were amplified by water molecules and detected by a gaseous secondary electron detectormounted at the tip of the objective.
